# The patient died: What about involvement in the investigation process?

**DOI:** 10.1093/intqhc/mzaa034

**Published:** 2020-05-14

**Authors:** Siri Wiig, Peter D Hibbert, Jeffrey Braithwaite

**Affiliations:** 1 SHARE Centre for Resilience in Healthcare, University of Stavanger, Norway; 2 Australian Institute of Health Innovation, Macquarie University, New South Wales; 3 Australian Centre for Precision Health, Cancer Research Institute, School of Health Sciences, University of South Australia, Adelaide, South Australia, Australia; 4 South Australian Health and Medical Research Institute, Adelaide, South Australia, Australia

**Keywords:** patient and family involvement, investigation, methods, research, practice

## Abstract

Patient and family involvement is high on the international quality and safety agenda. In this paper, we consider possible ways of involving families in investigations of fatal adverse events and how their greater participation might improve the quality of investigations. The aim is to increase awareness among healthcare professionals, accident investigators, policymakers and researchers and examine how research and practice can develop in this emerging field. In contrast to relying mainly on documentation and staff recollections, family involvement can result in the investigation having access to richer information, a more holistic picture of the event and new perspectives on who was involved and can positively contribute to the family’s emotional satisfaction and perception of justice being done. There is limited guidance and research on how to constitute effective involvement. There is a need for co-designing the investigation process, explicitly agreeing the family’s level of involvement, supporting and preparing the family, providing easily accessible user-friendly language and using different methods of involvement (e.g. individual interviews, focus group interviews and questionnaires), depending on the family’s needs.

## Introduction

Patient and family involvement is now seen to be of strategic importance in international quality and safety research and practice. However, it is unclear how families can be involved when a patient dies after suffering an adverse event and what kind of involvement we can expect from the many different healthcare investigatory bodies [1–6], ranging from internal investigations to independent national investigation boards. Here, we reflect on possible ways of involving families in investigations of fatal adverse events and how increased involvement potentially can improve investigations. Our aim is twofold: to generate ideas, reflection and critical discussion of how research and practice can help improve family involvement in investigations and spread information about the topic among healthcare professionals, accident investigators, policymakers and researchers.

## Investigations of fatal adverse events

We focus on consequential fatal adverse events caused by service provision or the lack thereof and not due to homicide. There is a growing interest in patient and family involvement in investigations and open disclosure [1–4], although examples of family involvement after patient deaths are less common [5, 6]. After fatal adverse events, different bodies may carry out investigations with varying purposes: root cause analysis (RCA) by service providers; legal investigations by regulatory bodies; learning-focused and system-oriented investigations by national investigation boards; public inquiries; and liability and prosecution by the police and court [7].

**Table 1:** Challenges in differing jurisdictions with family member involvement

Country	Potential challenges with jurisdictions
Norway	No major issue other than logistical and cultural. Specified and included in the NIBHC legislation
Australia	Differs by State but feasible federally and within State and territories
USA	Differs by State but potential civil liability, e.g. iatrogenic harm leading to death can inhibit family member participation

## What is a family?

The conceptual and operational definition of a ‘family member’ may differ in practice and research publications. In the literature, family members can be referred to as relatives in direct line but can also be referred to as a next of kin [5, 6, 8, 9]. Next of kin is, for example, according to Norway’s Patient and User Rights Act (1999) [10]: [§1.3B] defined by the patient or user and could be a close friend and not a direct relative. In cases when the patient or user is in a condition incapable of defining his or her next of kin, the order in the law starts with the spouse, registered partner, in-life partner, children over 18 years, parents, siblings over 18 years, grandparents or other family members who are in close relation with the patient or user [10]: [§ 1.3B]. Here we have chosen a broad use of the concept and integrate examples where studies have used both family members and next of kin to patients who have died in a fatal adverse event.

**Table 2:** Examples of novel investigative procedures

Type	Purpose	Utility
Interviews	• Collect information about the individual patient, adverse event, causal chain, involved healthcare personnel and stakeholders, and clarify questions	• Improved understanding of the adverse event and causal chain
		• Can be tailor made for type of fatal event (wrong site surgery, medication administration, events involving multiple stakeholders and system actors, contextual settings)
		• Contributes to doing justice to the family by allowing individuals to be heard and respected
		• Improved learning information in individual cases
		• Can be time-consuming and requires training of investigators
Focus groups	• Collect information about themes, experiences and patterns across different types of adverse event types, similar types of events, similar or different contextual settings or how to develop investigatory practice with family involvement	• Improved and tailor made procedures co-designed with families
		• Aggregated information with different types of themes and contexts that could inform areas of improvement activities and risk areas and inform accident prevention strategies
		• Needs training for investigators
Meetings	• Collect information or feedback information to individual or multiple family members in single cases of investigations. This could also work to discuss recommendations and dissemination of information	• Strengthens the interaction and communication between investigation body and the family
		• Strengthens the recommendations related to the investigation report
		• Allows individuals or families to be heard and respected
		• Can be time-consuming and emotionally challenging
		• Needs training for investigators
Questionnaires	• Collect information from individual cases where family members do not wish to participate in meetings and interviews or have disabilities or long travel distances	• Potential to contact difficult to reach groups and larger samples of family members
	• Collect information from groups of family members on, e.g. topics or themes of investigations, suggestions for dissemination activities or particular types of events	• Elicits information about attitudes and perspectives
		• Information is relatively objective
		• Low cost
		• Not time-consuming for investigators or family members as interviews or meetings and no travel needed
Conferences	• To disseminate findings to groups of families, healthcare professionals and policymakers	• Reaches wider audiences
	• To disseminate recommendations	• Spreads knowledge and facilitates information exchange
		• Can help build and restore trust in the system
		• Can be expensive and involves travelling for the audience and speakers
Consensus panel	• To develop recommendations	• Similar to many of the above utility aspects
		• Reaches a wider group of family members with experience with fatal adverse events to guide method development

## Why involve families in investigations?

Family members often have the most in-depth knowledge about patients, their health record, personal information and their journey through the health system [8, 9]. Some family members also have detailed knowledge about the fatal adverse event or causal factors, or both, that may provide rich information if they are involved as information sources. Even though this is an emerging area in different countries where new methods are being tested, usually family members are not involved as part of common investigation procedures or as part of the regulatory requirements associated with mandatory investigations [1–3, 5–6, 11].

Previous research provides examples where regulatory investigations experiment with incorporating family involvement via meetings and interviews with investigators [1, 5, 6]. This has reportedly sometimes resulted in improved investigation quality because the family gave investigators rich information, a more holistic picture of the fatal event and new perspectives about additional actors and stakeholders involved in the causality chain. Family involvement essentially contributes to closing the gap between ‘work as imagined’ (WAI) and ‘work as done’ (WAD) [5, 6]. Investigators often rely on written information exchange from healthcare professionals, organizations and technical information from medical technology, registries or records, while healthcare practice often differs, as observed and told by family members. Involving family members in interviews can thus contribute a depth of information compared with relying on written information exchange [5, 6]. Research from hospital internal investigations interviewing family members during data collection has found similar results. Family involvement contributed to the collection of valuable investigation information in the Netherlands and was adding to learning and clearly doing justice to those involved [12]. Family members who received an invitation to participate in regulatory investigations have also reported a positive therapeutic effect in being heard and can be appreciative of the opportunity to ask clarifying face-to-face questions of the investigators [5].

To exemplify what difference family involvement may constitute from an investigatory point of view, we note the experiences among investigators who tested the implementation of an involvement procedure in a project in Norway [5, 6]. In this project a 2-hour meeting between next of kin and regulatory investigators enabled next of kin to tell their version of the event, raise questions and inform investigators about aspects related to the fatal event and the deceased patient [5, 6]. The approach was used across the healthcare context, including fatal adverse events occurring in both primary care (e.g. nursing homes, homecare, general practitioners) and specialized care (hospitals, and in psychiatric care). It was also used to inform many types of cases such as suicide after discharge from institutions, cerebral hemorrhage, transfer of patients from hospital to nursing homes or transfer within hospital departments, falling in nursing homes and cancer [5:p 2]. The investigators involved indicated that the method provided them with much more relevant information than they had expected. Family members gave details about timeline, medication, history, symptoms, procedures, involved personnel and additional stakeholders, which sometimes differed compared to the written information from the service provider institutions or the involved healthcare personnel. The additional information helped investigators better establish the narrative and the picture of the event and its causal chain. When adding the information gathered from the meeting, this picture often changed [6].

The recently established independent Norwegian National Investigation Board for the Health and Care Services (NIBHC), similar to the English Healthcare Safety Investigation Branch, aims to have patient and family involvement at the heart of its activities [7]. Since May 2019, patients, users and next of kin, by law, have the right and opportunity to report adverse events to the NIBHC; to be informed about an investigation commencing; to make a statement about the event; and to comment on a draft report before publication [13]. For years, families who lost a close relative to a fatal adverse event had advocated for a learning-focused, independent investigation board where the patients’ and families’ perspectives would be taken seriously and integrated into investigation practice [7]. Although now law, it is yet to be seen how family involvement will be facilitated by NIBHC and influence its investigations.

In general, there are different jurisdictions and challenges with family member involvement across countries. In Table 1 we list some examples from Norway, Australia and the USA. This is not an exhaustive or detailed list but illustrates some potential challenges.

## Suggestions and challenges for practice improvement and research

It can be seen, then, that involving families in investigations has the potential to improve investigation quality by broadening perspectives and providing new learning information [1–2, 5, 6]. However, investigation bodies wishing to experiment with new methods of family involvement need to prepare for multiple challenges, such as meeting the investigation timeframe, emotional aspects, trust, epistemic injustice, possible conflicts of perspectives between healthcare providers and families and understanding of roles and the formal investigation process [5, 6, 12, 14]. Family members are in unique positions and constitute an important information source for investigators, but their routine involvement as we have seen is underutilized, and there is limited guidance and research available on what constitutes effective practice [4–6, 15, 16]. It has proven difficult sometimes for family members to understand the role, function, formalities, bureaucracy and work processes of investigation bodies and investigations into fatal events [5]. Therefore, good quality information with easily accessible user-friendly language should be provided to invited family members. Participation must be optional for a family because involvement can be traumatic and psychologically stressful [4–6]. There is a need to support and prepare a family if members wish to be involved and the level of their involvement needs to be explicitly agreed. By whom, when and how this support and preparation is offered and provided to families, needs further elaboration in most jurisdictions. From the research we have cited, it appears logical that involvement should commence early and the investigation may need to allow more time to be completed [2].

We need new knowledge about different methods of involvement, which may include individual interviews, focus group interviews, questionnaires and consensus panels. In Table 2 we illustrate examples of novel investigative procedures and indicate their potential purpose and utility. The list is not exhaustive and should be further expanded and evaluated.

An investigatory organization might have a range of these options depending on need, aims of the organization, the family’s expectations and agreed level of involvement, their requirements for support and the level of complexity of the fatal adverse event at issue. Research indicates that in some of the most complex fatal events involving actors across organizational interfaces in specialized healthcare and primary care, information from the families might be more central in investigations to understand causality compared with less complex events [6]. Research also shows that family members expect to be involved when patients die (complex causality or not) and, despite the potential for stress, can report positive experiences with the involvement, as we have seen [5]. Family involvement in the development of recommendations, or their dissemination, as well as contributing to a robust process for the investigatory organization, may provide a level of emotional satisfaction and epistemic justice for the family as they are potentially actively contributing to reducing the chance of the same event happening to another patient. In line with typical investigation methods, consensus panels may be the most appropriate method for involvement in the development of recommendations.

Almost all practice improvement and innovative investigation practices would benefit from co-development and co-designing with families, including their inclusion within the governance structures of the relevant investigatory organizations. Innovation and design of innovative involvement approaches should also incorporate iterative formative evaluations, gathering the views of families at the end of the process to ensure that their needs were met, risks were managed and improvements are embedded.

## Final reflections

Learning from fatal adverse events is fundamental for healthcare policy and practice and requires developing a repertoire with variety in methods, data sources and analytical perspectives [17]. We suggest that the field should develop and test investigation methods and practices that include procedures for consulting families and collecting data from family members from a range of sources including interviews, face-to-face meetings, questionnaires or focus group interviews. As indicated in Table 2, different methods have different uses and value and need to be considered on a case by case basis depending on causality, time frame, complexity of case and who is involved. Family members should be invited to suggest recommendations and be involved in their dissemination. This could also take different forms. In individual cases family members could have suggestions and input based on reading draft reports such as in the NIBHC’s practice [13]. We encourage experimentation with, and sharing of, new approaches.

The research community should be involved to evaluate and assess the impact of family involvement, e.g. in terms of improved investigation report quality; measurement of next of kin satisfaction with investigations and restorative practice; time to close regulatory investigations; implementation of recommendations; and reputation of investigatory bodies to mention only a few [5–6, 18]. We believe family involvement in investigation of fatal adverse events could realize untapped learning potential and encourage future research and evaluation to test and critically review new methods to tailor make them to fit both investigatory and family members’ needs for knowledge and improvement to prevent reoccurrences. Liability is also a topic that future research should focus attention on when exploring family involvement in investigations. Following on from Table 1, different countries and healthcare systems, legislation and actors such as legal personnel, insurers and regulators might directly or indirectly object to the involvement of family members as this could increase chances of lawsuits. This should be acknowledged when working towards practice changes involving families.
